# Management of Atypical Hangman’s Fracture (C2 Axis): Systematic Review of Classification, Treatment Strategies, and Clinical Outcomes

**DOI:** 10.3390/medicina62040637

**Published:** 2026-03-27

**Authors:** Stjepan Ivandić, Sathish Muthu, Lora Grbanović, Jay Toor, Jure Pavešić, Mišo Krstičević, Mirza Pojskić, Stipe Ćorluka

**Affiliations:** 1 Spinal Surgery Division, Department of Traumatology, University Hospital Centre Sestre Milosrdnice, 10000 Zagreb, Croatia; 2 Orthopaedic Research Group, Department of Spine Surgery, Coimbatore 641045, India; 3 Central Research Laboratory, Aarupadai Veedu Medical College and Hospital, Vinayaka Mission’s Research Foundation (Deemed to be University), Puducherry 607403, India; 4 Department of Radiology, University Hospital Center, 10000 Zagreb, Croatia; 5 Section of Orthopedics, Department of Surgery, University of Manitoba, Winnipeg, MB R3T 2N2, Canada; 6 Department of Neurosurgery, University of Marburg, 35037 Marburg, Germany; 7 St. Catherine Specialty Hospital, 10000 Zagreb, Croatia; 8 Department of Anatomy and Physiology, University of Applied Health Sciences, 10000 Zagreb, Croatia

**Keywords:** atypical hangman, C2 axis, management, systematic review, clinical outcomes

## Abstract

*Background and Objectives*: To provide a systematic narrative review of published literature on atypical hangman’s fractures focusing on pathophysiology, treatment options and clinical outcomes. *Materials and Methods*: A systematic review was performed according to PRISMA guidelines. MEDLINE (PubMed), EMBASE, Scopus, and Cochrane Library were searched until March 2025. Studies reporting outcomes of atypical hangman’s fractures treated conservatively or surgically were included. Data on demographics, mechanism of injury, treatment modality, outcomes, and complications were extracted and analyzed. *Results*: Thirteen studies with a total of 275 patients were included. The average age was 54.36 years. High-energy trauma was the predominant mechanism of injury. Conservative treatment was performed in 210 patients, with 204 (97.14%) achieving fusion and 6 (2.86%) converted to surgical treatment. Surgical fixation was performed in 71 patients, most commonly via a posterior approach. Failure of surgical treatment occurred in 5 patients, all treated with isolated anterior fusion. Neurologic injury was reported in 21 patients (7.63%), with full recovery in 14 (66%). *Conclusions*: Atypical hangman’s fractures represent a distinct subgroup of C2 fractures with diverse morphology and stability. Most fractures are stable and may be managed conservatively. Surgical fixation should be reserved for unstable patterns. If surgery is pursued, posterior fixation is recommended. Outcomes are generally favorable for both conservative and surgical treatment.

## 1. Introduction

Hangman’s fracture is a traumatic spondylolisthesis of the second cervical vertebra (C2) caused by bilateral pars fracture. Despite the name, hangman’s fractures are not usually associated with judicial hangings and are rarely fatal. Moreover, neurologic compromise is rare due to central canal widening from resulting anterolisthesis [1].

Most commonly utilized classifications are the Effendi [2] as well as the Edwards–Levine modification of the Effendi classification [3]. This distinguishes three different types of fracture morphology based on different mechanisms of injury, with Type I and some Type II usually considered stable [4,5]. These may be considered typical fracture patterns. In a subset of patients, the fracture pattern may be asymmetric and coronal, leading to axis body splitting in such a way that it behaves like a fracture dislocation due to intact bony ring [6]. This has an opposite effect on canal width compared with conventional hangman’s fractures, leading to increased risk of cord compression with anterior translation [4]. This leads to higher rates of neurologic injury compared with typical fractures. The key feature of this variant is fracture asymmetry. For the purposes of this review, atypical hangman’s fractures were defined as C2 body fractures with posterior wall involvement, distinct from typical pars fractures. Radiological criteria included fracture asymmetry, vertical orientation, or involvement of the posterior cortex. Ambiguous cases were adjudicated by consensus among reviewers.

Radiologic workup includes standard plain X-ray as well as CT and MRI, both of which are crucial for treatment algorithm [7]. Incidence varies between studies and has been reported to range between 18 and 74% [4]. Incidence assessment is unclear as these fractures may also be described as miscellaneous or axis body fractures. Additionally, atypical fractures could be considered as high-energy trauma due to overrepresentation in younger age group [8,9].

Treatment options are dependent on fracture stability. The Edwards–Levine classification may be utilized for atypical fractures. Li et al. developed a new complementary classification system dividing atypical fractures into A1, 12, B1 and B2, although it has yet to find widespread clinical use [10]. It has been suggested that fracture symmetry does not influence stability [11]. Surgical fixation is usually recommended for Types II, IIa and III and Type I with neurologic injury [10]. Conservative treatment options include hard cervical collar or halo vest. Due to complications associated with the halo vest and due to the lack of identified advantages, as demonstrated by the systematic review of Murphy et al., some recommendations omit halo vest immobilization in favor of the use of only a hard cervical collar [7,9,12]. In a study by Al-Mahfoudh et al., only 1 in 28 patients with atypical fractures required surgical fixation [13]. Based on their results, the authors recommend primary nonoperative management for all types of atypical fractures. An interesting difference in rates of surgical treatment, 37.5% vs. 76%, may be observed in two systematic reviews on management of hangman’s fractures published in 2006 [14] and 2017 [12].

Surgical treatment includes anterior cervical discectomy and fusion (ACDF), posterior spinal instrumentation and fusion (PSIF) and a combined anterior/posterior approach [4]. Another interesting and underutilized approach includes pars osteosynthesis with a Judet screw. It has been argued that ACDF may be an inappropriate surgical treatment method for atypical hangman’s as it may leave fracture gaps due to the inability to address the posterior part of the vertebral body [11,15]. Failure of ACDF requiring additional posterior fixation has been reported [16]. With respect to the typical hangman’s fracture, the recommendations of the spine section of the German Society for Orthopedics and Trauma propose treatment based on fracture dislocation and C2–C3 disc and anterior longitudinal ligament integrity [7].

Recently, two systematic reviews have provided a thorough analysis of treatment options on hangman’s fractures [12,15]. Atypical hangman’s may be considered a separate entity and currently no algorithm exists to guide treatment and the choice between surgical and conservative options remains a controversial one. The aim of this review is to provide a focused and comprehensive analysis of the published literature on atypical fractures and to provide a framework for the appropriate treatment protocol based on available evidence.

## 2. Materials and Methods

Systematic review was performed in accordance with PRISMA guidelines. The review was not prospectively registered and a formal review protocol was not published. Although the review was not prospectively registered in PROSPERO, an internal protocol was established prior to initiation. This protocol defined eligibility criteria, piloted screening and extraction procedures, and guided methodological decisions.

A comprehensive search was conducted in MEDLINE (PubMed), EMBASE, Scopus, and Cochrane Library until March 2025. Full search strings with Boolean operators and MeSH/Emtree terms are provided in Supplementary Table S1. Search dates and syntax specific to each database are reported to ensure reproducibility. Additional studies were handpicked from included studies, previous systematic reviews and narrative reviews if they fit inclusion criteria.

We included studies reporting on outcomes of atypical hangman’s fracture including conservative and/or surgical management, studies with clear and distinct atypical hangman’s case description or clearly stating “atypical hangman fracture,” and studies with fracture description consistent with the radiological definition of atypical hangman’s, with criteria including fracture asymmetry, vertical orientation, or involvement of the posterior cortex. We excluded studies including patients with hangman’s fracture without atypical variant, studies without clear fracture morphology description or not referring to the atypical variant, case reports, cadaver studies, finite element analysis and reviews. We also excluded studies in language other than English.

Once duplicates were removed, titles and abstracts were reviewed and screened using the online platform www.rayyan.ai. Screening was done independently by two authors (S.I. and S.M.). All retrieved records were imported into a reference management system, and duplicate entries were identified and removed using a two-step process. First, automated detection based on DOI, title, and author metadata was performed. Second, manual verification was conducted by two independent reviewers to ensure accuracy. Discrepancies were resolved by consensus. Eligibility criteria were finalized following pilot screening of 20 abstracts. Data extraction forms were piloted on three studies to refine variables and ensure consistency across reviewers. Data extraction was performed by the same authors. Data items included number of conservative and surgically treated patients, demographics, mechanism of injury, conservative treatment modality, type of surgical treatment, functional outcomes, neurologic injury, complications and failure of conservative or surgical treatment. Failure of conservative treatment was defined as conversion to surgical treatment. Failure of surgical treatment was defined as a need for revision surgery at the index level.

There was a significant variability in how functional outcomes were reported. This lack of uniformity hindered the ability to synthesize findings quantitatively or draw direct comparisons between studies. Studies that did not report on specific outcomes (e.g., neurologic injury) were still included for analysis if they provided other relevant data, given the limited available information on this topic. No assumptions were made for missing data. In some studies data extraction was severely limited due to combined outcomes and results for both typical and atypical hangman’s patient group. One study [17] was excluded because of concerns of overlapping datasets with one included study [10].

The methodological quality of the included studies was assessed using a modified version of the Newcastle–Ottawa scale (NOS), adapted for non-comparative observational studies [18]. The original NOS domains of selection, comparability, and outcome were retained, but the comparability domain was simplified as no control groups were available.

Selection (0–4 points): Representativeness of the cohort, clarity of inclusion criteria, ascertainment of exposure, and adequacy of follow-up.

Outcome (0–4 points): Method of outcome assessment, completeness of reporting, length of follow-up, and statistical analysis.

Overall methodological integrity (0–2 points): Transparency of study design and reporting.

Thus, the maximum possible score was 10 points. Studies scoring ≥ 7 were considered high quality, 5–6 moderate quality, and ≤4 low quality. Two reviewers independently applied the modified NOS, with disagreements resolved by consensus. The scoring criteria are detailed in Supplementary Table S2, and individual study scores are reported in Table 1.

Due to the heterogeneity of study designs, patient populations, and outcome reporting, a formal meta-analysis was not performed. Instead, data were synthesized using descriptive statistics. Continuous variables such as age and duration of treatment were summarized as means and ranges, while categorical variables such as mechanism of injury, treatment modality, complications, and neurologic outcomes were expressed as absolute frequencies and percentages. Where available, subgroup distributions (e.g., conservative vs. surgical treatment, anterior vs. posterior fixation) were tabulated to facilitate comparison.

## 3. Results

**Figure 1:**
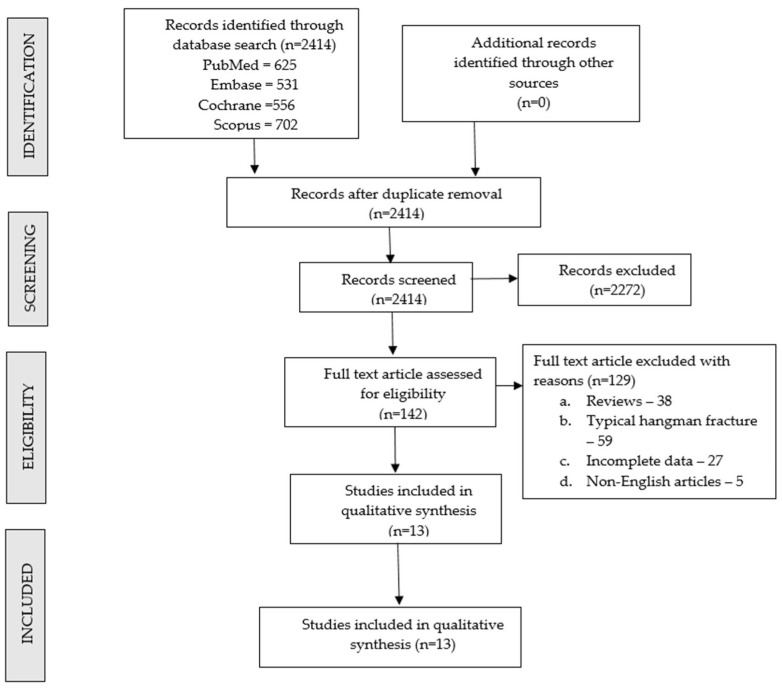
PRISMA flow diagram of inclusion of studies.

A total of 2414 records were screened; 142 were included for full text analysis, of which 13 were included for review. The study selection process is outlined in the PRISMA flow diagram (Figure 1), and a summary of the included articles with their methodological scoring is provided in Table 1. A total of 275 patients were included for analysis from the included studies, with a mean age of 54.3 years. Risk-of-bias assessment revealed that most studies were retrospective case series with limited outcome reporting. This influenced the strength of conclusions, which should be interpreted cautiously.

### 3.1. Mechanism of Injury

Mechanism of injury was reported in nine studies. The most common mechanism of injury includes motor vehicle accidents (reported in seven studies) followed by fall from height (reported in five studies) and other. In 148 patients, the mechanism of injury was high energy trauma. Preoperative MRI was reported to be obtained in eight studies [9,10,19,20,21,22,23,24]. MRI was assessed for disco-ligamentous injury and for spinal cord contusion or compression evaluation. In Niemeier et al., the authors reported that MRI did not provide additional data and showed no evidence of posterior longitudinal ligament of the C2–C3 disc [9], while Li et al. noted MRI to be utilized to assess stability based on C2–C3 disc and anterior longitudinal ligament integrity [10]. Neurologic injury was reported in 21/275 patients (7.63%). In two studies, the ASIA grade was utilized for grading [10,20], while in three studies descriptive grading was used [5,9,13]. Full neurologic recovery (stated as such and/or ASIA E) was reported in 14/21 patients.

### 3.2. Traction

Use of traction was reported in five studies [19,20,21,23]. Salunke et al. used a gentle preoperative traction using Crutchfield tongs with a weight of 2–3 kg [20]. Kyu et al. used traction preoperatively in six cases to reduce translation [21]. Aksan et al. used traction with a weight of 2–3 kg to all patients in the operating room [23].

### 3.3. Classification

**Figure 2:**
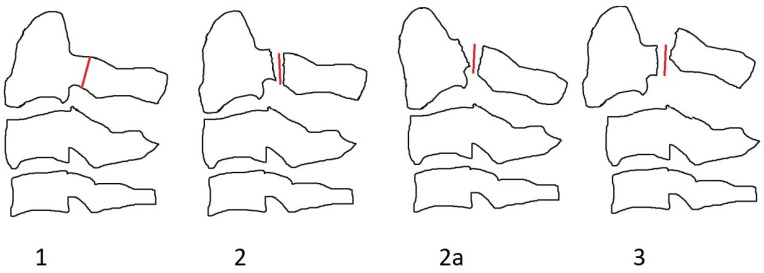
Levine and Edwards classification of hangman’s (C2) fractures (adapted from Aksan et al.).

**Figure 3:**
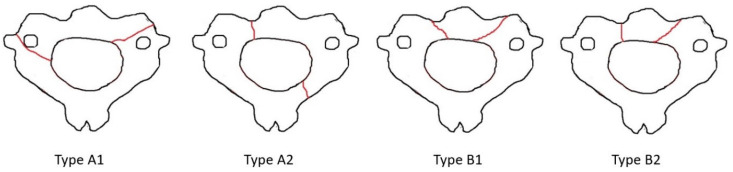
Li-Wang classification of atypical hangman’s fractures (adapted from Aksan et al.).

In nine studies, a classification system was reported [4,5,10,13,20,21,22,23,24]. In eight studies the Edwards–Levine classification (Figure 2) was utilized [4,5,10,13,20,21,22,23]. A new unique classification was proposed by Li et al. and was used by Aksan et al. [10,23] (Figure 3). A new classification was also reported by Al-Mahfoudh et al. [13]. In a study by German et al., atypical fractures were considered as Type I vertical body fractures [24]. In Robinson et al., Edwards–Levine was used for typical but not atypical hangman’s variants [8].

### 3.4. Conservative Treatment

Conservative treatment was reported in eight studies [4,5,8,9,10,15,22,24]. Two studies included only conservative management [9,24]. The total number of patients initially treated conservatively was 210, with six patients converted to surgical treatment after failure of conservative management. The total number of patients treated successfully conservatively was 204 (97.14%). Treatment modalities included hard cervical collar in 137 patients (7 studies), halo vest in 57 patients (6 studies), Minerva in 10 patients (1 study), and 1 unspecified [24]. All studies considered halo vest as a form of conservative treatment. Duration of treatment was specified in three studies. In a study by Al-Mahfoudh et al., duration of treatment was 117 days for halo and 124 days for cervical collar [13] and two other studies reported a three-month duration [5,24]. No study reported a detailed treatment protocol. The criteria for the conservative management outlined in the included studies were initial conservative treatment in all patients and surgical in cases of failure [8,13]; mild displacement defined as horizontal translation < 5 mm or C2–3 angulation < 15 degrees [9]; majority of Type I and some of Type II fractures [4,22]; and Type I of Edwards–Levine classification without neurologic deficit or soft tissue related instability [10].

Conservative management was typically chosen when the fracture was deemed stable. In five studies no conservative management was pursued [16,19,20,21,23]. Fusion was achieved in 97.14% (204/210) of initially conservatively treated patients. Duration of conservative treatment varied between studies, ranging from 2.5 months to 4 months. In a study by Botros et al., different conservative treatment modalities were chosen based on fracture classification. In cases of Edwards–Levine classification Type I, hard cervical collar is appropriate while invasive halo is recommended in cases of Edwards–Levine Type II variants [4]. In two studies, failure of conservative management was reported. Al Mahfoudh et al. reported one patient being converted to surgical treatment due to failed halo treatment [13]. Botros et al. reported five patients being converted to surgical treatment due to delayed fracture union [4].

### 3.5. Operative Treatment

Operative treatment was utilized in 11 studies. In five studies it was the sole treatment modality i.e., no conservative treatment was utilized [16,19,20,21,23]. The total number of patients treated surgically was 71. In three studies an additional postoperative cervical collar was used for 4–14 weeks [19,20,21].

### 3.6. Indications for Surgical Treatment

Indications for surgical treatment were outlined in all studies reporting on surgical treatment. In a study by Al Mahfoud et al., one patient was initially treated conservatively and converted to posterior fixation due to failure of non-operative treatment [13]. In a study by Botros et al., five patients were converted to surgery due to failure of conservative treatment and delayed healing (one patient in Edwards–Levine Type I, four in Edwards–Levine Type II). Authors concluded that both Edwards–Levine 1 and 2 may be initially treated conservatively, with either hard cervical collar or halo traction [4]. Kim et al. considered all teardrop variants of atypical hangman’s fractures to be unstable and requiring surgery [19]. Surgery was pursued for fractures deemed unstable (II, IIa and III), Type I fractures with neurological deficits and Type I fractures with associated soft tissue damage, ruptured anterior longitudinal ligament, and intervertebral disc at C2–C3, as per Li et al. [10]. In a study by Cai et al., 1 Edwards–Levine Type I and II and Edwards–Levine Type II patients were treated surgically [22]. Similarly, in a study by Aksan et al., Edwards–Levine Type I and the majority of Edwards–Levine Type II were considered stable, while IIa and III were deemed unstable [23]. In a study by Salunke et al., patients with Type II and IIa were deemed unstable and were surgically treated [20]. In a study by Kyu et al., the treatment method was based on patient preference, following a detailed explanation of the benefits and drawbacks of both treatment modalities. The disadvantages of conservative treatment, which include discomfort, residual neck pain, and reduced range of motion due to deformity were key factors associated with the decision to pursue conservative or surgical treatment [21]. In a study by Starr et al., only one patient with complete tetraplegia was surgically treated [5].

### 3.7. Functional Outcomes and Radiology

Reported functional outcomes vary widely between groups. Descriptive functional outcomes were reported by three studies [9,10,13]. In five studies, the VAS scale was used to compare preoperative and postoperative pain levels [10,19,20,21,23]. Al-Mahfoudh et al. reported 14% of patients with atypical fractures have moderate or severe pain and reported stiffness in 39% of patients [13]. Niemeier et al. reported universal good outcomes with either halo or hard cervical collar. All patients in their study were treated conservatively [9]. Li et al. found, among all patients at the final follow-up, that none had reported severe neck pain without evidence of neurologic deterioration and with solid bony fusion [10]. Reported radiological outcomes and fusion rates were assessed with different diagnostic modalities, including CT scans, standard anteroposterior and lateral radiographs and flexion and extension radiographs.

### 3.8. Complications

Two studies reported complications of conservative management. These include two pin-site infections, which were converted to hard collar and healed uneventfully [13] and one C2 angulated healing with local kyphosis but successful union [10]. Complications of surgical treatment were reported by three studies. In a study by Li et al., there was one esophageal perforation (combined approach), two severe bleeding (posterior) with one postoperative cerebellar ataxia, one pneumonia and one UTI [10]. Complications which resulted in failure of surgical treatment and need of revision were reported in two studies. In Botros et al. [4], additional posterior fixation for two ACDFs after 26 days for residual instability was required and, in a study by Aljubori et al. [16], all three patients required revision surgery. All failures of surgical treatment occurred in patients undergoing an isolated anterior approach. Mortality was reported in two studies. Botros et al. [4], and German et al. [24] reported 17.6% (9/51) and 6.6% (1/15) mortality among their included patients, respectively.

Failure of surgical treatment was defined as a need for revision surgery because the initial treatment goals were not achieved. Failure of surgical treatment was reported in two studies [4,16]. Failure of anterior approach was reported in five patients. In a study by Botros et al., two patients treated with ACDF required additional posterior fixation after 26 days due to residual instability [4]. In a study by Aljubori et al., all three patients treated with ACDF required revision and posterior fixation due to pseudoarthrosis [16]. Failure of posterior approach was reported in none of the included patients.

## 4. Discussion

Atypical hangman’s fractures are a rare subset of axis spondylolisthesis injuries. This systematic review highlights their distinct morphology, stability patterns, and management strategies. Most fractures were associated with high-energy trauma, yet neurologic injury was relatively uncommon (7.6%) and often reversible. Conservative treatment achieved fusion in 97% of cases, with very low conversion to surgery. Surgical fixation was performed in 71 patients, with posterior approaches showing superior reliability compared with isolated anterior fusion, which had higher failure rates. Overall, both conservative and surgical strategies yielded favorable outcomes when appropriately selected.

Although several binary outcomes were reported, heterogeneity in fracture definitions, treatment thresholds, and reporting standards precluded statistical pooling. For example, criteria for ‘failure of conservative treatment’ varied across studies. Therefore, a narrative synthesis was chosen to avoid misleading pooled estimates.

### 4.1. Terminology and Morphology

Uniform description of fracture morphology remains problematic. The term “atypical hangman” generally refers to C2 body fractures involving the posterior wall, visualized as Edwards–Levine types I–III with posterior wall involvement. Fracture lines may traverse the posterior wall rather than the pars, forming a closed circle. Other terms include “hangman’s variant” or “vertical body fracture.” Benzel et al. described sagittal and vertical body fractures as involving the body proper, without conventional spondylolisthesis [25]. Burke and Harris defined variants as separation of the axis body from the posterior arch with vertically oriented defects involving the posterior cortex [9,26]. Some authors have avoided treatment recommendations due to heterogeneity [8].

### 4.2. Neurologic Injury

Historically, atypical fractures were thought to carry higher neurologic risk due to canal narrowing (“closed ring” hypothesis). Starr et al. coined the term in 1993, reporting neurologic injury in 33% (2/6) [5]. Larger series reported lower rates: Botros et al. none and Li et al. 12/46 (26%) [4,10]. In this review, overall incidence was 21/275 (7.63%), with recovery in 14/21 (66%). Lower incidence may reflect variability in posterior wall continuity; bilateral detachment may increase canal compromise. High-energy trauma may also cause subaxial cord injury, explaining deficits not directly related to C2. Starr et al. did not use MRI, possibly overestimating C2 involvement. Most authors consider atypical fractures to be relatively stable, with lack of comminution and displacement favoring fusion [24]. Mechanism is likely axial loading with extension [25]. Stability remains debated. Older criteria such as White and Panjabi, the Francis variant, and Roy-Camille may be applied [11]. Li et al. have suggested that atypical fractures arise from similar forces as typical hangman’s but with added rotational components, producing asymmetry. Bilateral posterior cortex fractures may compromise the canal, with vertical compressive forces resisted by C2/C3 facets [10].

### 4.3. Classification

Several systems have been used. Most studies applied Edwards–Levine, though atypical fractures differ from typical C2 body injuries. Li et al. have proposed a unique classification tailored to atypical patterns [10], complementary to Edwards–Levine. Al-Mahfoudh also reported a new system [13]. These remain limited in validation and scope, with criticisms that coronally oriented fractures may represent axis body injuries rather than hangman’s variants [10].

### 4.4. Conservative Treatment

Edwards–Levine Type I and most Type II fractures were considered stable and treated conservatively. Some authors have recommended nonoperative care for all atypical fractures [8,13]. Our review found low complication rates, with only 6/220 patients (2.72%) converted to surgery. Al-Mahfoudh reported one failed halo case requiring posterior fixation [13]. Niemeier et al. treated 63 patients conservatively (<5 mm translation or <15° angulation) with 100% union [9]. Conservative modalities included halo and hard collar. No clear guidelines distinguished their use. Halo traction remains debated, particularly in elderly patients due to complications (12–36%) such as pin-site infection, abscess, falls, and respiratory issues [13]. Halo offers fracture reduction [27]. Treatment duration ranged from 2.5 to 4 months [4,5,9,13,24].

### 4.5. Surgical Treatment

Indications varied but generally included neurologic injury, Edwards–Levine Type III, and unstable Type II/IIa fractures. Surgical options were poorly standardized [28]. Posterior fixation was most common, including direct pars osteosynthesis, C2–C3, C1–C3, or occipitocervical fusion. Posterior fixation is favored for correcting kyphosis and avoiding flexion deformity [14]. Li et al. performed C1–C3 arthrodesis due to vertebral artery anatomy and small C2 pedicle [10]. Motion-preserving direct osteosynthesis at C2 maintains segmental mobility. Kyu et al. used minimally invasive tubular retractors, reporting full range of motion and reduced pain at 6 months [21]. Indications are limited; osteosynthesis may be inadequate for >4 mm translation or disc injury [13,20]. Some argue isolated C2 fixation is unnecessary, as stable fractures may respond to conservative care [28]. Posterior fixation addresses fracture fragments and soft tissue instability, allowing reduction of angular listhesis [20]. Disadvantages include inability to treat intercalated disc fragments, risk of stenosis, or forward displacement of C2 [29].

### 4.6. Anterior vs. Posterior Approaches

Failures were more frequent with anterior fixation. In this review, 5/7 anterior cases failed. Salunke et al. noted the technical challenges of anterior C2–3 fusion due to C2 curvature, difficulty correcting angulation, and inadequate apposition [20]. Murphy et al. found no differences in union, complications, or mortality between ACDF, posterior, or combined approaches [12]. ACDF offers disc access, reduced blood loss, and less tissue injury [13]. Biomechanical studies support posterior fixation. Duggal et al. showed that posterior C2–C3 fixation provided greater stability than anterior plating, especially in lateral bending and rotation [30], functioning like a tension band [31]. Conversely, Arand et al. argued anterior plating is mandatory in traumatic spondylolisthesis with C2–C3 instability [32]. Combined anterior–posterior fixation was reported in four patients [4,10], usually after anterior failure. Occipitocervical fixation was reported in one patient [5], but its utility is limited by loss of cervical motion. Mahmoud’s review concluded posterior C2–C3 fusion is most appropriate, arguing that ACDF inadequately stabilizes the posterior arch [13].

### 4.7. Proposed Management Algorithm

At admission, X-ray, CT, and MRI should be performed to establish fracture morphology and soft tissue status. Classification using Edwards–Levine is an appropriate first step. MRI should then be scrutinized for disc integrity and severe ligamentous injury. In cases of incomplete or complete neurologic deficit, immediate surgical decompression is recommended according to current AO spine guidelines, ideally within the first 24 h [33]. For severe instability, surgery should be performed as early as feasible.

For Edwards–Levine Type I fractures, a non-operative trial is appropriate. Follow-up radiographs should be obtained at 2 and 4 weeks. Immobilization with a hard cervical collar for 10–14 weeks is generally sufficient, though duration should be individualized. CT may be used at final follow-up to confirm fusion. The role of halo traction is limited and may be omitted due to associated complications.

For Edwards–Levine Type II and IIa fractures, a tailored approach is required. Conservative treatment is recommended if translation is <3 mm and kyphosis > 15°/lordosis > 5°. Either conservative or surgical management may be considered for translation of 3–5 mm and kyphosis 15–20°. Definitive surgical fixation is indicated if translation exceeds 5 mm with kyphosis > 20°.

When surgery is pursued, a posterior approach is preferred, consistent with prior systematic reviews [13]. In minimally displaced fractures (angulation < 20°, displacement < 4 mm), direct osteosynthesis may be considered if expertise and resources are available. If displacement exceeds these thresholds or if C2–C3 instability is present (ligamentous or disc injury), posterior C2–C3 fixation is appropriate. Conventional pedicle or pars screws should be used as laminar screws are often inadequate due to atypical morphology. An anterior approach may be considered in cases of significant disc herniation.

**Figure 4:**
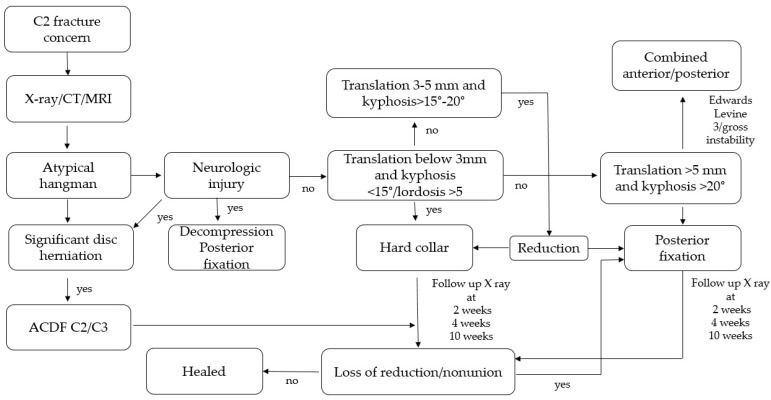
Proposed management algorithm.

If anatomy precludes safe C2 fixation or if expertise is limited, C1–C3 fixation should be performed. Occipitocervical fixation remains a last-resort option for highly unstable multilevel injuries but must be weighed against the drawback of restricted neck motion. Preoperative skull traction may be routinely employed to aid reduction. Patients may be discharged after brief hospitalization if no other injuries are present. Recommended follow-up includes visits at 2 weeks, 4 weeks, 3 months, 6 months, and 1 year. The proposed algorithm is summarized in Figure 4.

The management algorithm presented here represents a synthesis of available literature and expert consensus. It is intended as a hypothesis-generating framework rather than a validated guideline. Thresholds for surgical indications (translation > 5 mm, kyphosis > 20°) were derived from criteria reported in included studies [9].

### 4.8. Limitations

This systematic review has several important limitations. First, the majority of included studies were retrospective in nature, which introduces the risk of selection bias and incomplete datasets. Second, most studies did not report patient-reported outcomes, limiting the ability to assess functional recovery and quality of life. Third, due to the paucity of literature on atypical hangman’s fractures, several lower-quality studies were necessarily included, which may affect the overall strength of evidence. Fourth, the definition of atypical fractures varied considerably across studies, raising the possibility that relevant reports were excluded because fracture morphology was inadequately described. Furthermore, in some studies, data specific to atypical fractures could not be separated from typical variants, further restricting the precision of pooled analysis. The most significant limitation of this study is the attempt to narratively synthesize the available evidence on a rare fracture subtype using studies with high heterogeneity. Considerable variability exists with respect to fracture subtypes, indications for surgical versus conservative treatment, and different treatment modalities. This variability significantly limits the ability to draw definitive conclusions regarding the optimal strategy. Nevertheless, consistent trends across the included studies have been observed, such as the high success rate of conservative treatment, the successful utilization of the Edwards–Levine classification, and the relatively low incidence of neurologic injury. However, these findings should be interpreted with caution.

## 5. Conclusions

This systematic review is the first to specifically address the pathophysiology, treatment strategies, and complications associated with atypical hangman’s fractures. These injuries most commonly result from axial loading and extension, often with a variable rotational component. The majority of fractures are stable and can be managed conservatively, with neurologic injury being relatively rare. Treatment decisions should be guided by the Edwards–Levine classification. Conservative management, typically with a hard cervical collar or, less commonly, halo immobilization, achieves high fusion rates. When surgery is indicated, posterior fixation (C2–C3 fusion or direct osteosynthesis in minimally displaced cases) is the preferred approach. The anterior route has limited utility and should be reserved for Edwards–Levine Type III fractures or cases with significant disc herniation. Overall, both conservative and surgical modalities yield favorable outcomes when appropriately selected, underscoring the importance of individualized, classification-based treatment planning.

## Figures and Tables

**Table 1:** Articles included in the review (NR—not reported, MVC—motor vehicle collision, ACDF—anterior cervical discectomy and fusion, MIS—minimally invasive screws, VAS—visual analogue scale, ROM—range of motion, NOS—Newcastle–Ottawa scale).

Study	Number of Patients (M/F)	Mechanism	Mean Age (Years)	Conservative (Type/N)	Surgical (Type/N)	Complications (N)	Failure	Functional Outcomes	Neurologic Outcomes (N)	NOS Score
Al-Mahfoudh	28 (NR)	Falls, MVC	NR	Halo (21), collar (6)	Posterior fixation (1)	Pin-site infection (2)	1 conservative converted to surgical	Moderate pain. Stiffness	Focal neuro-recovered (3)	3
Robinson	24 (NR)	NR	61	Halo (2), collar (20)	NR (2)	NR	NR	NR	NR	4
Niemeier	63 (NR)	High-energy trauma	55	Halo (15), collar (48)	0	NR	No progressive displacement	NR	Complete neurologic injury (1)	3
Botros	51 (21/30)	MVC, falls	66	Halo (13), collar (27)	Posterior fixation (8), ACDF (1), combined (2)	Death (9)	Additional posterior fixation for 2 ACDFs 5 failures of hard collar converted to surgical	NR	0	5
Kim	6 (3/3)	Falls, slips, MVC	37	0	ACDF (2), posterior fixation (4)	0	None	VAS improvement	0	3
Li	46 (36/10)	Falls, MVC, other	47	Halo (27), collar (19)	Posterior fixation (20), arthrodesis (4), ACDF (1), combined (2)	Esophageal perforation (1), severe bleeding (2), cerebellar ataxia (1), pneumonia (1), UTI (1)	1 nonoperative C2 angulated healing with local kyphosis	No complaints of severe neck pain at final follow up	12 (2 ASIA C, 1 ASIA B, 9 ASIA D), all recovered 1 or 2 grades	5
Salunke	6 (4/2)	MVC, falls	46	0	Posterior fixation (6)	NR	NR	VAS	1 ASIA D	4
Man Kyu	7 (5/2)	MVC, falls	51	0	MIS screw using tubular retractors (7)	0	None	VAS improvement, full ROM at 6 months	0	5
Cai	16 (NR)	MVC, falls	NR	Collar (13)	3 NR	NR	NR	NR	0	4
Starr	6 (4/2)	MVC	31	Halo (5)	Occipitocervical fusion (1)	NR	None	NR	2 (1 complete C3 quadriplegic, 1 incomplete C3 hemiplegia), second patient recovered, ambulatory at 6 months	5
Aksan	4 (NR)	NR	NR	0	Posterior fixation (4)	NR	NR	NR	NR	4
German	15 (9/6)	MVC	47	Minerva (10), collar (4), halo (1)	0	Death (3)	NR	Symptoms resolved, no pain	1 incomplete unrelated	4
Aljubori	3 (2/1)	NR	59	0	ACDF (3)	NR	Pseudoarthrosis	NR	0	4

## Data Availability

The original contributions presented in this study are included in the article/Supplementary Material. Further inquiries can be directed to the corresponding author.

## Supplementary Materials

The following supporting information can be downloaded at https://www.mdpi.com/article/10.3390/medicina62040637/s1, Supplementary Material: Reporting checklist for systematic review—Tables S1 and S2.

## Abbreviations

The following abbreviations are used in this manuscript:

CT	Computed tomography
MRI	Magnetic resonance imaging
PRISMA	Preferred Reporting Items for Systematic Reviews and Meta-Analyses
NOS	Newcastle–Ottawa scale
ACDF	Anterior cervical discectomy and fusion
PSIF	Posterior spinal instrumented fusion
MIS	Minimally invasive surgery
MVC	Motor vehicle collision
UTI	Urinary tract infection
VAS	Visual analog scale
ROM	Range of motion
ASIA	American spinal injury association

## References

[1] Schleicher P., Scholz M., Pingel A., Kandziora F. (2015). Traumatic Spondylolisthesis of the Axis Vertebra in Adults. Glob. Spine J..

[2] Effendi B., Roy D., Cornish B., Dussault R.G., Laurin C.A. (1981). Fractures of the ring of the axis. A classification based on the analysis of 131 cases. J. Bone Joint Surg. Br..

[3] Levine A.M., Edwards C.C. (1985). The management of traumatic spondylolisthesis of the axis. J. Bone Joint Surg. Am..

[4] Botros M., Singh A., Shaikh H., Ramirez G., Molinari R.W., Puvanesarajah V. (2025). Atypical Hangman’s Fractures: An Institutional Study of 51 Patients with Atypical Traumatic Spondylolisthesis of C2. Glob. Spine J..

[5] Starr J.K., Eismont F.J. (1993). Atypical hangman’s fractures. Spine.

[6] Turtle J., Kantor A., Spina N.T., France J.C., Lawrence B.D. (2020). Hangman’s Fracture. Clin. Spine Surg. Spine Publ..

[7] Scholz M., Kandziora F., Kobbe P., Matschke S., Schleicher P., Josten C., the Spine Section of the German Society for Orthopaedics and Trauma (2018). Treatment of Axis Ring Fractures: Recommendations of the Spine Section of the German Society for Orthopaedics and Trauma (DGOU). Glob. Spine J..

[8] Robinson A.L., Möller A., Robinson Y., Olerud C. (2017). C2 Fracture Subtypes, Incidence, and Treatment Allocation Change with Age: A Retrospective Cohort Study of 233 Consecutive Cases. BioMed Res. Int..

[9] Niemeier T.E., Manoharan S.R., Mukherjee A., Theiss S.M. (2018). Conservative Treatment of Hangman Variant Fractures. Clin. Spine Surg. Spine Publ..

[10] Li G., Zhong D., Wang Q. (2017). A novel classification for atypical Hangman fractures and its application: A retrospective observational study. Medicine.

[11] Samaha C., Lazennec J.Y., Laporte C., Saillant G. (2000). Hangman’s fracture: The relationship between asymmetry and instability. J. Bone Joint Surg. Br..

[12] Murphy H., Schroeder G.D., Shi W.J., Kepler C.K., Kurd M.F., Fleischman A.N., Kandziora F., Chapman J.R., Benneker L.M., Vaccaro A.R. (2017). Management of Hangman’s Fractures: A Systematic Review. J. Orthop. Trauma.

[13] Al-Mahfoudh R., Beagrie C., Woolley E., Zakaria R., Radon M., Clark S., Pillay R., Wilby M. (2016). Management of Typical and Atypical Hangman’s Fractures. Glob. Spine J..

[14] Li X.F., Dai L.Y., Lu H., Chen X.D. (2006). A systematic review of the management of hangman’s fractures. Eur. Spine J..

[15] Mahmoud A., Shanmuganathan K., Montgomery A. (2023). Surgical Management of Hangman’s Fracture: A Systematic Review. Int. J. Spine Surg..

[16] Aljuboori Z., Hoz S., Boakye M. (2020). Failure of C2-3 anterior arthrodesis for the treatment of atypical Hangman’s fractures: A three case series. Surg. Neurol. Int..

[17] Li G., Wang Q., Liu H., Hong Y. (2018). Individual Surgical Strategy Using Posterior Lag Screw–Rod Technique for Unstable Atypical Hangman’s Fracture Based on Different Fracture Patterns. World Neurosurg..

[18] Luchini C., Stubbs B., Solmi M., Veronese N. (2017). Assessing the quality of studies in meta-analyses: Advantages and limitations of the Newcastle Ottawa Scale. World J. Meta-Anal..

[19] Kim S., Rhee J.M., Park E.T., Seo H. (2021). Surgical Outcomes for C2 Tear Drop Fractures: Clinical Relevance to Hangman’s Fracture and C2-3 Discoligamentous Injury. Orthop. Surg..

[20] Salunke P., Karthigeyan M., Sahoo S.K., Prasad P.K. (2018). Multiplanar realignment for unstable Hangman’s fracture with Posterior C2-3 fusion: A prospective series. Clin. Neurol. Neurosurg..

[21] Man Kyu C., Youngseok K., Ki Hong K., Dae-Hyun K. (2019). Direct trans-pedicular screw fixation for atypical hangman’s fracture: A minimally invasive technique using the tubular retractor system. J. Clin. Neurosci..

[22] Cai Y., Khanpara S., Timaran D., Spence S., McCarty J., Aein A., Nunez L., Arevalo O., Riascos R. (2022). Traumatic spondylolisthesis of axis: Clinical and imaging experience at a level one trauma center. Emerg. Radiol..

[23] Akşan Ö., Seçer M. (2023). Surgical Management of the Hangman’s Fracture in the Twelve Cases: Case Series. Turk. Klin. J. Med. Sci..

[24] German J.W., Hart B.L., Benzel E.C. (2005). Nonoperative management of vertical C2 body fractures. Neurosurgery.

[25] Benzel E., Baldwin N.G. (1994). Fractures of the C-2 vertebral body. J. Neurosurg..

[26] Burke J.T., Harris J.H. (1989). Acute injuries of the axis vertebra. Skelet. Radiol..

[27] Vaccaro A.R., Madigan L., Bauerle W.B., Blescia A., Cotler J.M. (2002). Early Halo Immobilization of Displaced Traumatic Spondylolisthesis of the Axis. Spine.

[28] Prost S., Barrey C., Blondel B., Fuentes S., Barresi L., Nicot B., Challier V., Lleu M., Godard J., Kouyoumdjian P. (2019). Hangman’s fracture: Management strategy and healing rate in a prospective multi-centre observational study of 34 patients. Orthop. Traumatol. Surg. Res..

[29] Liu J., Li Y., Wu Y. (2013). One-stage posterior C2 and C3 pedicle screw fixation or combined anterior C2-C3 fusion for the treatment of unstable hangman’s fracture. Exp. Ther. Med..

[30] Duggal N., Chamberlain R.H., Perez-Garza L.E., Espinoza-Larios A., Sonntag V.K.H., Crawford N.R. (2007). Hangman’s Fracture: A Biomechanical Comparison of Stabilization Techniques. Spine.

[31] Ma W., Xu R., Liu J., Sun S., Zhao L., Hu Y., Jiang W., Liu G., Gu Y. (2011). Posterior short-segment fixation and fusion in unstable Hangman’s fractures. Spine.

[32] Arand M., Neller S., Kinzl L., Claes L., Wilke H.J. (2002). The traumatic spondylolisthesis of the axis A biomechanical in vitro evaluation of an instability model and clinical relevant constructs for stabilization. Clin. Biomech..

[33] Kwon B.K., Tetreault L.A., Evaniew N., Skelly A.C., Fehlings M.G. (2024). AO Spine/Praxis Clinical Practice Guidelines for the Management of Acute Spinal Cord Injury: An Introduction to a Focus Issue. Glob. Spine J..

