# Supplementary material for: Management of Atypical Hangman’s Fracture (C2 Axis): Systematic Review of Classification, Treatment Strategies, and Clinical Outcomes
Source: Medicina (Kaunas). 2026 Mar 27;62(4):637. doi: 10.3390/medicina62040637 (PMC13117135; doi:10.3390/medicina62040637)
Supplement: Supplementary file 1 [file medicina-62-00637-s001.zip › Supplementary Table S1.pdf]

Supplementary Table S1. Detailed Search Strategy for Systematic Review

Database	Search String / Syntax	Controlled Vocabulary (MeSH/Emtree)	Limits Applied	Date of Search
**PubMed (MEDLINE)**	(“Hangman fracture” OR “atypical Hangman” OR “Hangman variant” OR “axis fracture” OR “C2 fracture” OR “traumatic spondylolisthesis of the axis”) AND (“treatment” OR “management” OR “surgery” OR “conservative”)	MeSH terms: *Axis fractures*, *Cervical vertebrae/injuries*, *Spinal fractures/surgery*	Humans, English language	March 15, 2025
**EMBASE**	(‘hangman fracture’ OR ‘atypical hangman’ OR ‘axis fracture’ OR ‘c2 fracture’ OR ‘traumatic spondylolisthesis’) AND (‘treatment’ OR ‘management’ OR ‘surgery’ OR ‘conservative’)	Emtree terms: *Axis fracture*, *Cervical spine injury*, *Spinal fixation*	Humans, English language	March 15, 2025
**Scopus**	TITLE-ABS-KEY (“Hangman fracture” OR “atypical Hangman” OR “axis fracture” OR “C2 fracture” OR “traumatic spondylolisthesis”) AND TITLE-ABS-KEY (“treatment” OR “management” OR “surgery” OR “conservative”)	Not applicable	Humans, English language	March 15, 2025
**Cochrane Library**	(“Hangman fracture” OR “atypical Hangman” OR “axis fracture” OR “C2 fracture” OR “traumatic spondylolisthesis”)	MeSH terms: *Cervical spine fracture*, *Spinal injuries*	Humans, English language	March 15, 2025

- Boolean operators (AND/OR) were used consistently across databases.
- Both free-text keywords and controlled vocabulary (MeSH/Emtree) were applied where available.
- No date restrictions were applied; all records up to March 15, 2025 were included.
- Reference lists of included studies and relevant reviews were hand-searched to identify additional eligible articles.
