# Supplementary material for: Management of Atypical Hangman’s Fracture (C2 Axis): Systematic Review of Classification, Treatment Strategies, and Clinical Outcomes
Source: Medicina (Kaunas). 2026 Mar 27;62(4):637. doi: 10.3390/medicina62040637 (PMC13117135; doi:10.3390/medicina62040637)
Supplement: Supplementary file 1 [file medicina-62-00637-s001.zip › Supplementary Table S2.pdf]

Supplementary Table S2. Modified Newcastle–Ottawa Scale (NOS) for Non-Comparative Observational Studies

Domain	Criteria	Score Range	Description
**Selection**	Representativeness of cohort; clarity of inclusion criteria; ascertainment of exposure; adequacy of follow-up	0–4	One point awarded for each criterion met.
**Outcome**	Method of outcome assessment; completeness of reporting; length of follow-up; appropriateness of statistical analysis	0–4	One point awarded for each criterion met.
**Overall Methodological Integrity**	Transparency of study design; clarity of reporting	0–2	One point awarded for each criterion met.
**Total Score**	—	0–10	Higher scores indicate better methodological quality.

Interpretation of Scores:

- **High quality:** ≥7 points
- **Moderate quality:** 5–6 points
- **Low quality:** ≤4 points
